# Oscillometric pulse wave analysis for detecting low flow arteriovenous fistula

**DOI:** 10.1186/s12882-023-03243-w

**Published:** 2023-06-24

**Authors:** Veit Busch, Joachim Streis, Sandra Müller, Niklas Mueller, Felix S. Seibert, Thomas Felderhoff, Timm H. Westhoff

**Affiliations:** 1 Nephrovital, Kamen, Germany; 2 Fachhochschule Dortmund, Dortmund, Nordrhein-Westfalen, Germany; 3 Pleiger Maschinenbau GmbH & Co KG, Witten, Germany; 4 Technische Universität Wien Institut für Diskrete Mathematik und Geometrie, Vienna, Vienna, Austria; 5 Klinikum der Universität München, Medizinische Klinik und Poliklinik III, Munich, Bavaria, Germany; 6 Department of Internal Medicine I, University Hospital Marien Hospital Herne, Ruhr-University Bochum, Bochum, Germany

**Keywords:** Pulse wave analysis, Haemodialysis, arteriovenous fistula, Duplex sonography

## Abstract

**Background:**

Pulse wave analysis may be useful to assess fistula function. We aimed to prospectively evaluate if convenient oscillometric devices are applicable to detect flow below 500 ml/min in a real life clinical setting.

**Methods:**

Pulse waves were recorded ambilaterally with the vicorder® device at the brachial artery in 53 patients on haemodialysis with native fistula. Primary variables consisted of the mean slope between the systolic maximum and the diacrotic notch (Slope2), the sum of the mean slopes in the four characteristic sections of pulse waves (Slope∑) and the amplitude of relative volumetric change in the measuring cuff at the upper arm (AMP). Fistula flow was measured with the use of duplex sonography using a standardized approach.

**Results:**

Parameter values above or below the median indicated measurement at the non-fistula side, with sensitivities/specificities of 0.79/0.79 (*p* < 0.001) for Slope 2, 0.64/0.64 (*p* = 0.003) for Slope∑ and 0.81/0.81 (*p* < 0.001) for AMP if measurements at the fistula and non-fistula arm were considered. ROC-analyses of parameter values measured at the fistula to detect low flow demonstrated AUCs (with CI) of 0.652 (0.437–0.866, *p* = 0.167) for Slope2, 0.732 (0.566–0.899, *p* = 0.006) for Slope∑ and 0.775 (0.56–0.991, *p* = 0.012) for AMP. The point with maximal youden’s index was regarded as optimal cut-off, which corresponded to sensitivities and specificities of 0.8/0.56 for slope2, 0.86/ 0.56 for Slope∑ and 0.93/0.78 for AMP.

**Conclusion:**

Functional surveillance with oscillometry is a promising clinical application to detect a low fistula flow. Among all investigated pulse wave parameters AMP revealed the highest diagnostic accuracy.

**Graphical Abstract:**

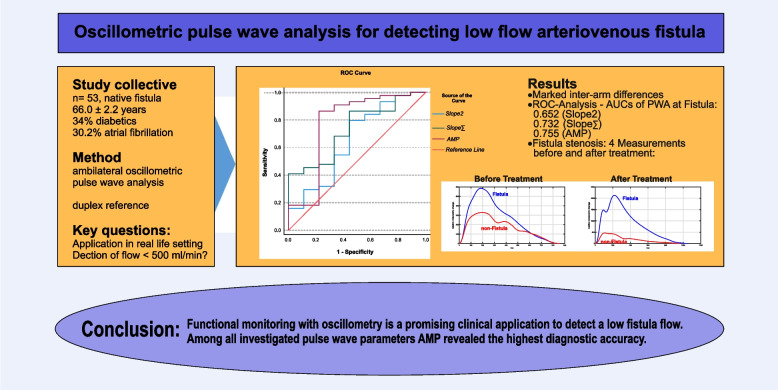

## Background

Peripheral pulse wave analysis (PWA) is an established tool to non-invasively assess vascular function [1–3], mainly in terms of vascular stiffness and estimating pressure in the ascending aorta and its relation to time during cardiac cycle [4, 5]. We recently demonstrated that PWA in proximity of forearm arterio-venous fistulas (AVF) may also characterize the function of AVFs [6], which serve as vascular access for haemodialysis and—from a haemodynamic point of view—induce high flow in the adjacent vessels [7]. Using tonometric measurement we demonstrated that the contours of pulse wave in arms with AVF differed significantly from those of the contralateral arm [6]. It remains elusive, however, whether PWA can be suitable as an easy tool to detect fistula dysfunction in daily clinical routines.

AVFs are the preferable type of vascular access for most patients on haemodialysis but there is a substantial burden of complications [8–10]. As such, most often AVF-stenosis occurs which causes low blood flow and significantly predisposes to AVF occlusion [11, 12]. Fistula flow-surveillance to preemptively detect AVF-stenosis may allow treatment before the occurrence of access failure [13–15] and potentially even to assess systemic vascular health [16]. Duplex sonography is an established method for that purpose [7]. Nevertheless, both procedures, i.e. tonometric PWA and duplex sonography depend on operator training and are quite time consuming [17, 18].

We therefore aimed to evaluate whether a more convenient method of PWA like oscillometry may be applied to evaluate fistula function, especially to detect low shunt flow as an indicator of stenosis.

## Methods

### Study enrollment and protocol

The presented prospective observational clinical study included haemodynamically stable patients on haemodialysis with native fistula at the forearm or at the upper arm. Patients with non-occluded fistula at contralateral arm, prosthetic arteriovenous grafts or central venous catheters were excluded. All measurements took place during a haemodialysis procedure in the outpatient Diavital dialysis unit in Kamen or in the Marien Hospital Herne, Ruhr-University Bochum, and consisted of non-invasive local consecutive ambilateral brachial PWA. Duplex sonographic measurements in the A. brachialis in both arms were performed to assess fistula blood flow. Four patients were treated for fistula stenosis during the study and in those patients we performed measurements before and after treatment of fistula stenosis. The study was approved by the local ethics committee of the Ruhr-Universität Bochum (No.15–5279) and participants provided informed consent which was written. Recruitment of patients took place between November 19^th^ 2019 and June 2021 26^th^.

### Pulse wave analysis and measurement of pulse wave velocity

**Fig. 1:**
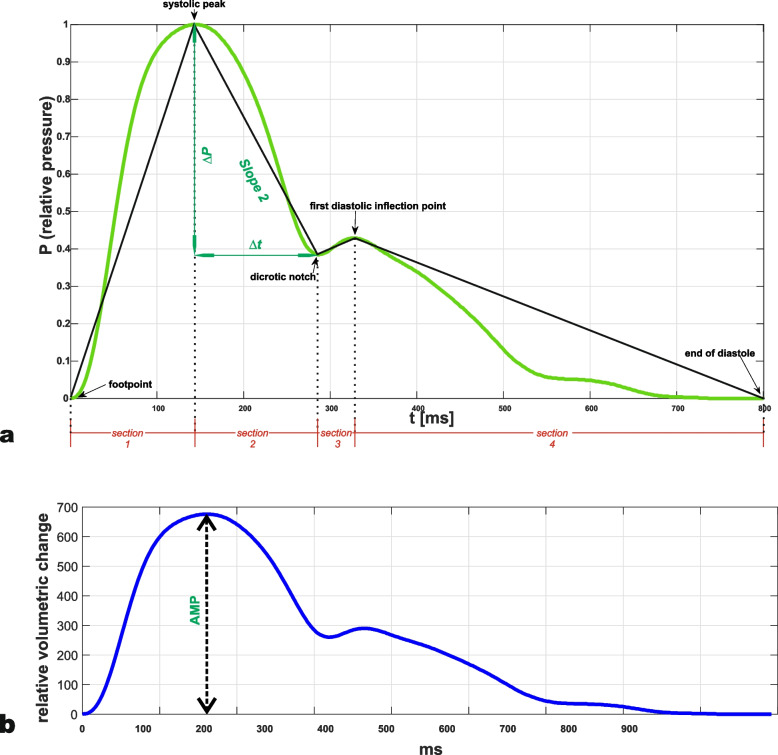
Illustration of the analysed pulse wave parameters Visualization of the key parameters by an exemplary pulse wave (average of beats recorded in 7 seconds, non-fistula arm, patient No 44). In panel** a** the curve is normalized to a relative pressure amplitude of 1 and a wave length of 800 ms. Slope parameters are calculated as the mean slope in the characteristic four time sections as defined by the footpoint, the systolic peak, the dicrotic notch, the first diastolic inflection point and the end of diastole (time sections are illustrated in red, the characteristic points are indicated with black arrows, the mean slope is visualized by black straight lines between the characteristic points). The variable Slope2 is the mean slope in the second time section and calculated as the ratio of the increments of relative pressure ∆P and time ∆t (green straight lines). Likewise, the mean slopes are computed in time sections one, three and four. Thereafter Slope∑ is calculated as the sum of the four mean slopes. In panel** b** the same measurement is presented before normalization**.** The y-axis shows the volumetric change in the measuring cuff in order to maintain a constant cuff pressure of 10 mmHg below end diastolic blood pressure as compensation for bloodpressure changes during cardiac cycle. According to Boyle-Mariotte's law the values of volumetric change are directly proportional to the bloodpressure changes during cardiac cycle which are assessed by pulse wave analysis. AMP is the amplitude of the non-normalized averaged pulse wave.

Oscillometric PWA was performed with the Vicorder® device (Skidmore Medical, Bristol, UK via SMT Medical, Würzburg, Germany). It allows to record brachial pressure waveforms by untrained medical stuff: A cuff is placed in the middle of the upper arm. During the measurements a pressure of 10 mmHg below the diastolic bloodpressure to safely prevent AVF-thrombosis can be applied automatically by the device and pulse waves are assessed with a volume displacement technique [19, 20]. Beats were averaged by the device during a measuring period of seven seconds, which according to the manufacturer allows to compensate for variations of wave length and wave amplitudes, e.g. in case of atrial fibrillation. For illustration, an exemplary averaged pulse wave is presented in Fig. 1.

### Duplex sonography

Volume flow (*Vf* in [ml/min]) was the main duplex variable (assessed as the product of the crosssectional area of the A. brachialis and mean flow velocity). A low flow fistula was defined by Vf < 500 ml/min as is supported by clinical data [13]. Duplex sonography was performed with the Toshiba® Xario 200 device in Kamen and the Siemens® ACUSON P500 device in Herne applying a high frequency (7–10 MHz) linear 38 mm probe.

### Evaluation algorithm and analyzed parameters

We programmed a MATLAB® environment to import and analyze digitized curves recorded with and averaged by the Vicorder® device. Pulse waves were normalized to a cycle length of 800 ms and an amplitude of 1. Following previous work, we calculated parameters in the four sections of the pulse wave between the wave footpoint, the systolic maximum, the dicrotic notch, the first diastolic inflection point and the end of diastole (as defined by measurement at the nFi-arm) in order to characterize AVF’s impact on pulse waveform [6]. The key parameters consisted of the mean slope in section two (*Slope2* in [relative amplitude/sec]) and the sum of the mean slopes (*Slope∑ in* [relative amplitude/sec]) in the four sections. Moreover, we calculated the amplitude of the non-pressure calibrated wave signal as exported from the Vicorder® device (*AMP*, [relative volumetric change]) as a co-primary parameter (which unlike the slope parameters was extracted from non-normalized curves). AMP is a measure of the peak of the displaced volume in the pressure detecting cuff in the course of the systole. In preliminary measurements prior to the presented study, differences in AMP between the fistula and non-fistula arm were so striking, that we decided to amend AMP to the other prespecified variables. The principles for calculation of the key parameters are visualized in Fig. 1, also see [6].

The location of the measurement site at the fistula or non-fistula arm (Status *Fi* versus *nFi*) is indicated by a lowercase index, e.g. Vf_Fi_, and the difference of each parameter between both arms by the prefix *∆*, e.g. ∆Slope2.

In case of measurements before and after treatment of fistula stenosis we calculated treatment induced increments of values measured at the fistula arm as well as of inter-arm-differences.

More mathematical backgrounds of the assessment of digitized wave curves are presented elsewhere [6].

### Statistical analysis

Standard univariate statistical analyses were used for description of demographic and clinical parameters.

To demonstrate the differences of pulse wave morphology at the fistula and non-fistula arm, we compared the medians of parameter values taken at fistula arm with the corresponding values taken at the non-fistula arm by application of the related-samples Wilcoxon signed rank test.

We then performed a pooled analysis of measurements at the fistula and non-fistula arm, reasonably assuming that the non-fistula arm can be regarded as a model for a non-functioning fistula: Parameter values were classified as *high/low* according to be above or below the median of all measurements (*overall median*), irrespective whether a value was obtained at the fistula or non-fistula arm. The high/low status was considered to be a simple test to predict on which side the value was taken assuming that in a case of a correct classification also a non-functioning fistula would be detected. Thereafter, sensitivities and specificities of the *low/high* class to detect *Fi/nFi*-status were calculated and one sided fisher’s exact test applied.

Finally, we focused on detection of low flow fistulas: Statistical ROC-analysis was used to quantify the pulse wave parameters’ diagnostic capacity to detect *low flow* fistulas, as assessed by duplex only at the fistula arm. We analysed parameter values taken at the fistula arm, as well as differences of values taken at fistula arm and the corresponding values taken at the non-fistula arm. The results are presented as specific AUC with 95% confidence interval and associated two-sided p-value. In a second step, maximal Youden-indexes and corresponding sensitivities, specifities, positive and negative predictive values were calculated. Significance refers to local, unadjusted *p*-value < 0.05 (< 0.0167 considering the Bonferroni correction, assessing three variables). Statistical analyses were performed using IBM SPSS Statistics for Windows, Version 27.0.

## Results

### Study population

We analyzed 53 patients. Before, one patient had to be excluded from analysis because of insufficient measuring quality and another because of the impossibility to place the cuff at the upper arm due to the position of dialysis needles in an upper arm fistula. Clinical characteristics are reported in Table 1. Mean age was 66.0 ± 2.2 years, mean dialysis vintage 43.2 ± 6.9 months and mean body mass index 25.6 ± 0.6 kg/m2. 38 (71.7%) were male, 30 (56.6%) diabetics, 18 (34%) suffered from confirmed coronary heart disease, 9 (17%) from confirmed peripheral arterial occlusive disease, 5 from heart failure with reduced ejection fraction (9.4%), 8 (15.1%) from right heart failure and/or pulmonary hypertension and 16 (30.2%) from permanent atrial fibrillation. 36 (67.9%) of fistulas were located on the left hand side and 37 (69.8%) at the forearm. Low fistula flow was documented in 9 patients (prevalence of 0.179).

**Table 1:** Clinical characteristics

**N**	53 (100%)
Age [years]	66 ± 2.2
BMI [kg/m2]	25.6 ± 0.6
Dialysis vintage [months]	43.2 ± 6.9
Gender [m/f]	2.53
Fistula side [left/right]	2.12
Fistula location [forearm/upper arm]	2.31
Fistula´s age [months]	43.3 ± 6.2
Diabetes	30 (56.6%)
Hypertension	50 (94.3%)
Permanent atrial fibrillation	16 (30.2%)
PAOD	9 (17%)
CHD	18 (34%)
HFrEF	5 (9.4%)
HFpEF	24 (45.3%)
Right HF/PH	8 (15.1%)

PAOD peripheral aterial occlusive disease, *CHD* Coronary heart disease, *HFrEF* Heart failure with reduced ejection fraction, *HFpEF* Heart failure with preserved ejection fraction, *HF* Heart failure, *PH* Pulmonary hypertension

### Fistula versus non-fistula status

The medians of Slope2, Slope∑, AMP, Vf and the cross-sectional area of the brachial artery significantly differed between the fistula and non-fistula arm (Table2).

**Table 2:** Comparison of fistula and non-fistula measurements

						**Overall Median**	**Fi versus nFi status**		
**Parameter**	**Median**_**Fi**_ (*n* = 53)	min/max	**Median**_**nFi**_ (*n* = 53)	min/max	**p** (Wilcoxon)	(Fi and nFi, *n* = 106)	**Sensitivity**	**Specificity**	**p (**Fisher's exact**)**
**Slope2** [relative amplitude/ms]	-0,0015	-0.00408/ 0.00497	-0,00322	-0.00564/ -0.00183	< 0.001	-0,00269	0,79	0,79	< *0.001*
**Slope∑** [relative amplitude/ms]	0,01094	-0.00022/ 0.01383	0,01187	0.00893/ 0.01666	0,006	0,01146	0,64	0,64	*0,003*
**AMP** [relative volumetric change]	1850	473/ 5236	725	190/ 2180	< 0.001	1030	0,81	0,81	< *0.001*
**Vf** [ml/min]	795	247/ 2128	58	2/ 168	< 0.001	207	1	1	< *0.001*
**A**_**brach.**_ [mm^2^]	32	16/ 71	19	9/ 44	< 0.001	25	0,7	0,72	< *0.001*

Lower case index indicating fistula (Fi) and non-fistula (nFi) measurements, for discription of variables see method section of the main document

In the pooled analysis of measurements at the fistula and non-fistula arm, it became apparent, that all values of Vf above the overall median were taken at the fistula arm, whereas all values of Vf below the overall analysis were taken at the non-fistula arm.

For Slope2, Slope∑ and AMP *high* values (i.e. above the overall median) may have been taken both at the fistula or the non-fistula arm. The analysis of (not depicted) fourfold tables with *Fi/nFi* status versus *high/low* parameter values was significant for all parameters (p: fisher's exact in Table 2) and sensitivities and specificities for parameter’s overall median to predict Fi and nFi-status calculated from those fourfold tables are presented in Table 2 (because of the consideration of measurements at both arms n was 106 for this illustrative analysis).

### ROC-analysis of low flow shunt

The AUC for all PWA parameters were numerically above 0.6 for measurements at the fistula arm as well as for Fi-nFi-differences. A statistical significance of ROC-analysis was shown for Slope∑_Fi_, AMP_Fi_ and ∆AMP. Details of the ROC-analysis are presented in Table 3. The maximum of youden indexes for each parameter was considered as optimal cut-off. Sensitivities, specificities, positive and negative predictive values for those cut-off values are listed in Table 3. For exploration also the ratio AMP_Fi_/ AMP_nFi_ was analysed, showing an AUC of 0.848 (confidence interval 0.695–1.002, *p* < 0.001).

**Table 3:** Testing for low flow fistulas: ROC-Analysis

Parameter	AUC	p	Lower Bound	Upper Bound	maximal Youden's-Index	corresponding cut-off	Sensitivity	Specificity	Positive predictive value	Negative predictive value
Slope2_Fi_	0,652	0,167	0,437	0,866	0,35	0,000026	0,8	0,56	0,27	0,93
∆Slope2	0,624	0,253	0,411	0,836	0,28	0,002744	0,73	0,44	0,21	0,89
Slope∑_Fi_	*0,732*	*0,006*	0,566	0,899	0,42	0,009639	0,86	0,56	0,29	0,95
∆Slope∑	0,662	0,163	0,434	0,889	0,32	0,000782	0,61	0,78	0,36	0,91
AMP_Fi_	*0,775*	*0,012*	0,56	0,991	0,64	1138	0,86	0,78	0,44	0,96
∆AMP	*0,823*	*0,002*	0,622	1,024	0,71	416	0,93	0,78	0,46	0,98

For discription of variables see method section of the main document, ROC receiver operator curve, presented by AUC and confidence interval bounds with *p*-value, *n* = 53: Values of measurements at the fistula arm (Slope2_Fi_, Slope∑_Fi_ and AMP_Fi_) as well as differences of measurements at the fistula and non-fistula arm (∆Slope2, ∆Slope∑ and ∆AMP) were analysed. A low flow fistula was defined by duplex measurements of flow < 500 ml/min at the fistula arm

### Measurements before and after treatment of shunt-stenosis

**Fig. 2:**
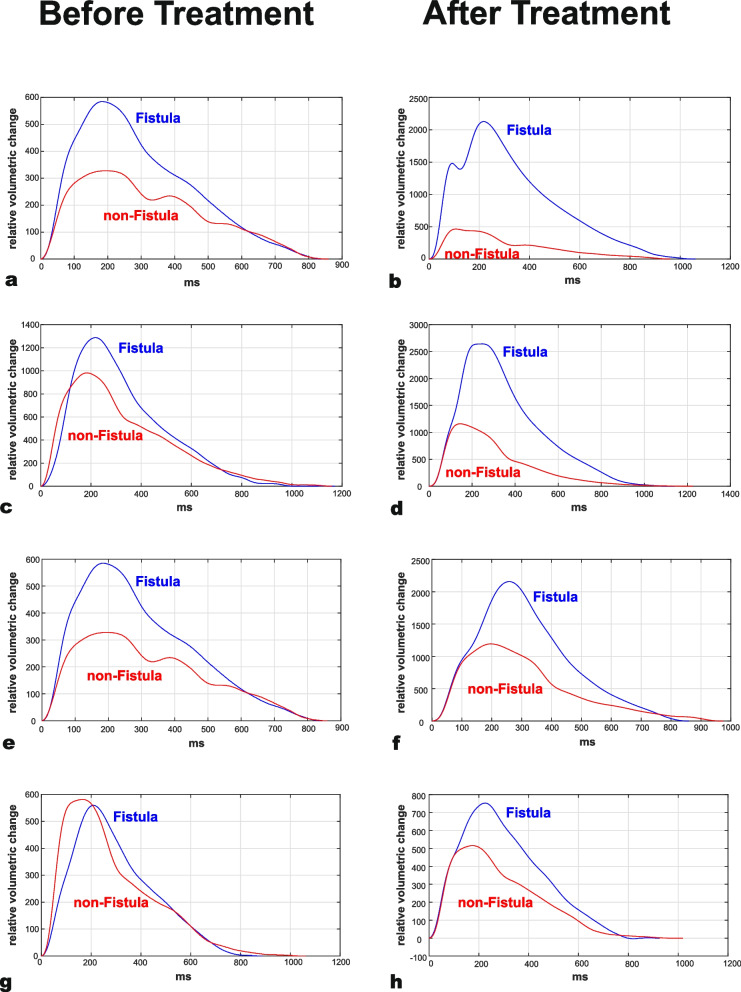
Pulse waves before and after treatment of shunt-stenosis Pulse waves as assessed by uncalibrated measuring cuff volume substitute in the course of the cardiac cycle (unnormalized cycle length) of four patients before (left panels **a**, **c**, **e** and **g**) and after (right panels **b**, **d**, **f** and **h**) treatment of fistula stenosis, curves of measurements at the fistula arm and non-fistula arm

**Fig. 3:**
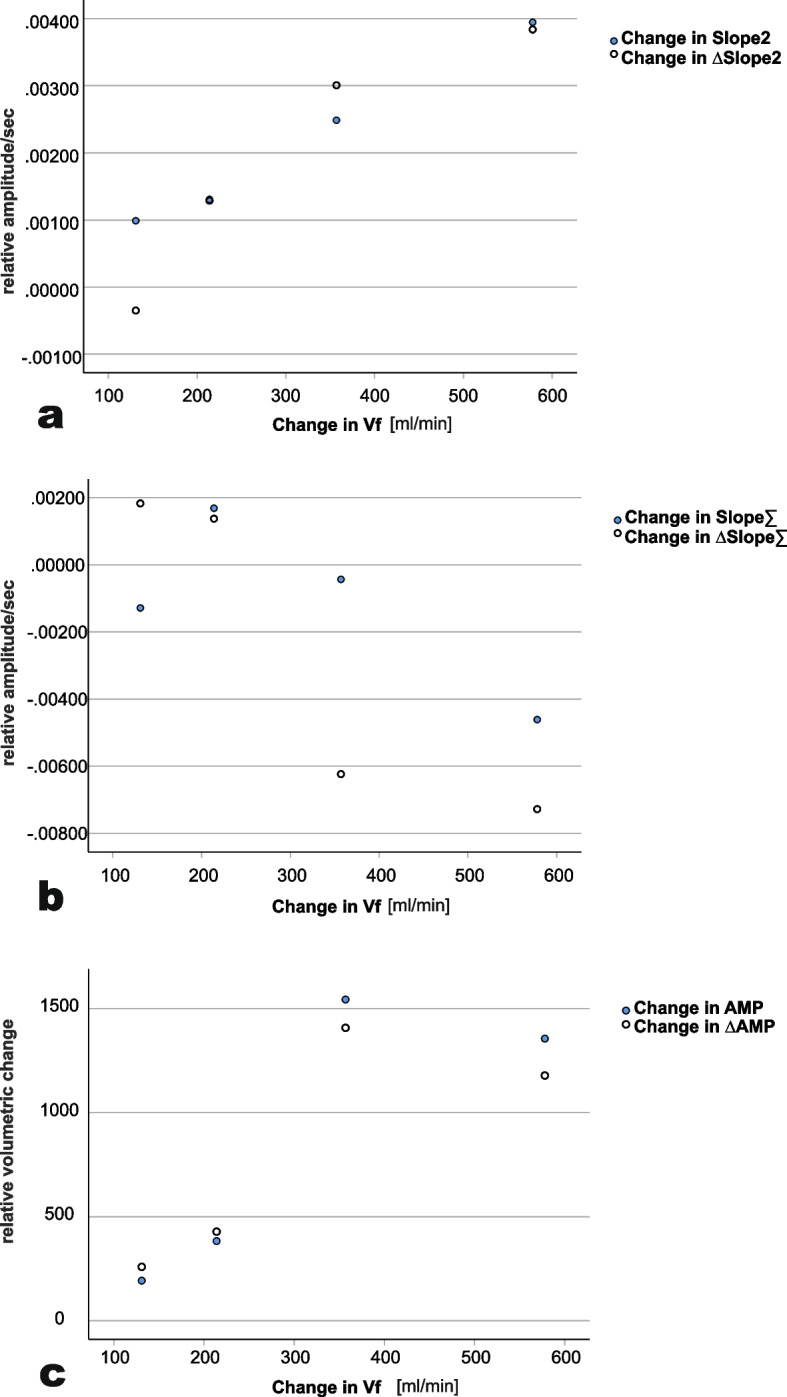
Change in pulse wave parameters versus change in volume flow Change of pulse wave variables in relation to change in fistula volume flow Vf [mL/min] after treatment of fistula stenosis, Slope2 and Slope∑ in [relative amplitude/ms], AMP [dimensionless], filled circles for measurements at the fistula arm, unfilled circles for differences between fistula and non-fistula arm

Pulse wave parameters differed before and after treatment, as is shown in Fig. 2. In Fig. 3 the relation of the change in Vf and the change in PWA-parameters and their Fi-nFi-differences by treatment of the fistula stenosis is visualized. Despite the small number of measurements those findings may indicate a proportional relationship as hypothesis.

## Discussion

In this cross-sectional prospective observational study, we were able to confirm that oscillometric PWA is a feasible option for fistula surveillance and can be useful to screen for low flow. From a perspective of practical application, oscillometry has several advantages in comparison to both duplex sonography and tonometric PWA: The required time for operator training and the inter-observer variability are neglectable [21]. What is more, it can be applied easily and expeditiously [17, 22]. Therefore, cost-savings might be reasonably assumed.

Our study was performed in a real life clinical setting with measurements during haemodialysis and included patients with forearm and upper arm AVF. The average age of 66 years and the considerable comorbidities underline that the method is applicable on a broad scale of nephrological routine.

The clinical characteristics were different in our previous pilot study with tonometric measurements, whose participants consisted mainly of kidney transplant recipients at an average age of 55 years not relying on haemodialysis nor having atrial fibrillation and solely of patients with forearm fistula [6]. The differential clinical characteristics also imply that the arteries of the presented study in comparison to the pilot study were more stiff [23–25]. This has an impact on pulse wave form [1] without apparent limitation in the presented area of application.

In a mixed analysis of parameter values taken at the Fi and nFi arm all *high* Vf values were taken at the Fi arm, whereas *high* values of the PWA parameters were more irregularly distributed between both arms (Table 2). This classification can be considered as a simple diagnostic test under the reasonable assumption, that in case of extreme fistula dysfunction, pulse waves at the Fi and nFi arm are not distinguishable, i.e. that parameters taken at the Fi-arm under those circumstances should be classified as nFi, and it proved that duplex sonography is a suitable reference method (Table 2), but also pulse wave analysis seems to be useful to indicate non-functioning fistulas.

In clinical practice the detection of low flow before occurrence of severe dysfunction is more desirable [15] and the analysis of high and low parameters considered measurements at both arms, whereas the focus should be on the measurements at the fistula arm. In order to examine the clinical utility of the novel parameters, we performed ROC-analyses for measurements at the fistula arm and for parameter inter-arm differences to detect fistula flow above/below a cut-off of 500 ml/min, which suggested that PWA indeed may serve as a screening test to detect low flow AVF (Table 3). This is underlined by negative predictive values well above 0.9 (Table 3). The comparatively low positive predictive values indicate, that a confirming test might be necessary.

We could assess parameters before and after treatment of shunt-stenosis in four patients. In those measurements, we demonstrated an increase in Vf which was paralleled by according changes in PWA parameters and their inter-arm differences, respectively (Fig. 3). This is in line with the hypothesis that alterations in pulse waveform as assessed by the presented slope parameters and AMP probably can indicate fistula stenosis which is haemodynamically relevant, although only few measurements before and after treatment of fistula stenosis were performed.

The question arises, whether measurements only at the fistula arm are sufficient or if ambilateral measurement is superior. Our results varied by regarded parameter and type of analysis (Table 3, Fig. 3). Given, that in previous analysis of tonometrically generated data only parameter differences of ambilateral measurements were impactful and from a theoretical point of view the inter-arm comparison may compensate for confounding, we would suggest that in future trials PWA should be performed at the Fi and nFi arm. Especially in case of atrial fibrillation, it even would be preferable to record pulse waves simultaneously at the Fi and nFi-arm, which was impossible when performing our study.

Approximately 30% of the patients suffered from permanent atrial fibrillation which can increase pulse frequency and amplitude variations and therefore reduce data quality and increase confounding by asynchronous measurements. Permanent atrial fibrillation was more prevalent in our study than in larger crosssectional studies of patients on haemodialysis (30.2% versus 3.5–10.7%) [26, 27]. We had not defined atrial fibrillation as an exclusion criterion, since this is not desirable with respect to clinical utility. Moreover, applicability of PWA in patients with atrial fibrillation has been demonstrated [28, 29] and beat-to-beat variability in wavelength and amplitude is mitigated both by averaging beats over 7 s and by normalization of amplitude and wavelength (the latter only for the slope-parameters).

According to our analyses, AMP may be regarded as the most promising parameter and technically its assessment is quite simple. Nevertheless, there is a lack of pressure calibration because non-invasive blood pressure measurement is impossible at the fistula arm. Given that AMP in contrast to the slope parameters is not normalized and that the arterial blood pressure has more influence on the amplitude than on the slope of pulse waves [1] this is an important issue. To compensate for those circumstances it may be valuable to assess the non-dimensional AMP Fi/nFi ratio in future trials, which according to a secondary analysis of the presented measurements at both arms seems to be a useful parameter. Nevertheless, AMP is a relative, dimensionless parameter and therefore values measured with the Vicorder® may not simply be compared with those measured with distinct devices. Having in mind that AMP relies on the volumetric change in the upper arm cuff during the temporal course of a pulse wave, it has to be postulated that not only the pulsations in the conducting artery but also in the fistula vein are impactful. This is a possible explanation for the strong differences in AMP at the fistula and non-fistula arm and furthermore for the exemplarily demonstrated increase of AMP after treatment of fistula stenosis (Figs. 2, and 3). Therefore, the recording of AMP may be designated as arteriovenous plethysmography.

Vascular cross-sectional areas and thus presumably compliance was greater at the Fi than at the nFi arm (Table 2), which theoretically leads to an increased Windkessel-function [30] and reduced vascular stiffness [31, 32], which is typically seen in the large central arterial vessels [1] and which may be regarded as a possible explanation for Vf dependent PWA-parameter differences of the fistula and non-fistula arm. Moreover, in case of peripheral arteries with reduced elasticity pulse waves have a gain of high-frequency components due to weaker damping, whereas in case of an increased vascular compliance damping of high frequency components may be enhanced [6, 33], which is a complementary approach to analyze the observed effects and diagnostic properties of the pulse wave parameters.

Our study has some limitations, which have not been completely discussed. The proposed mechanism should be analysed in more detail, e.g. by wave separation [34, 35]. Ideally, in future trials differences in impedance of the fistula and non-fistula arm should be assessed by simultaneous recording of pressure and flow, like Collard et al. did to assess intra-glomerular pressure [36]. The number of included patients was 53, but they were heterogeneous in terms of age, chronic heart failure and, as already discussed, atrial fibrillation, all of which may have an impact on pulse wave morphology [1]. However, the possibility of comparison of measurements at the fistula and non-fistula arm, the averaging of beats during seven seconds and the normalization of amplitude and wavelength may at least compensate partly for heterogeneity. Given that patients were included at two different locations, a confounding by the distinct ultrasound devices at the sites cannot be totally precluded.

In future, a broader clinical evaluation is of great importance: The four measurements before and after treatment of fistula stenosis indicate systematic measurement before and after treatment of shunt-stenosis as a possibly suitable trial design. Nevertheless, it is still under debate, in which cases pre-emptive treatment of AVF-stenosis is clinical useful [37]. That is why, also longitudinal studies and randomized controlled clinical trials with clinical outcomes are desirable.

## Conclusion

We augmented the evidence that PWA may be useful to assess AVF-function, especially in terms of detection of low fistula flow.

## Data Availability

The datasets used and analysed during the current study as well as the applied matlab™ code to assess digitized pulse waves are available from the corresponding author on reasonable request.

## Abbreviations

Slope2: Mean slope between the systolic maximum and the diacrotic notch
SlopeΣ: Sum of the mean slopes in the four characteristic sections of pulse waves
AMP: Amplitude of relative volumetric change in the measuring cuff at the upper arm
PWA: Pulse wave analysis
AVF: Arterio-venous fistula
Vf: Volume flow
Fi: Fistula arm
nFi: Non-fistula arm
PAOD: Peripheral arterial occlusive disease
CHD: Coronary heart disease
HFrEF: Heart failure with reduced ejection fraction
HFpEF: Heart failure with preserved ejection fraction
HF: Heart failure
PH: Pulmonary hypertension
